# Oxidative Stress in Aging: Advances in Proteomic Approaches

**DOI:** 10.1155/2014/573208

**Published:** 2014-02-13

**Authors:** Daniel Ortuño-Sahagún, Mercè Pallàs, Argelia E. Rojas-Mayorquín

**Affiliations:** ^1^Laboratorio de Desarrollo y Regeneración Neural, Instituto de Neurobiología, Departamento de Biología Celular y Molecular, CUCBA, Universidad de Guadalajara, Camino Ing. R. Padilla Sánchez 2100, Las Agujas, Zapopan, 44600 Jalisco, JAL, Mexico; ^2^Unitat de Farmacologia i Farmacognòsia Facultat de Farmàcia, Institut de Biomedicina (IBUB), Centros de Investigación Biomédica en Red de Enfermedades Neurodegenerativas (CIBERNED), Universitat de Barcelona, 08028 Barcelona, Spain; ^3^Departamento de Ciencias Ambientales, Instituto de Neurociencias, CUCBA, Universidad de Guadalajara, 45100 Jalisco, JAL, Mexico; ^4^Departamento de Investigación Básica, Instituto Nacional de Geriatría (INGER), Periférico Sur No. 2767, Colonia San Jerónimo Lídice, Delegacion Magdalena Contreras, 10200 México, DF, Mexico

## Abstract

Aging is a gradual, complex process in which cells, tissues, organs, and the whole organism itself deteriorate in a progressive and irreversible manner that, in the majority of cases, implies pathological conditions that affect the individual's Quality of Life (QOL). Although extensive research efforts in recent years have been made, the anticipation of aging and prophylactic or treatment strategies continue to experience major limitations. In this review, the focus is essentially on the compilation of the advances generated by cellular expression profile analysis through proteomics studies (two-dimensional [2D] electrophoresis and mass spectrometry [MS]), which are currently used as an integral approach to study the aging process. Additionally, the relevance of the oxidative stress factors is discussed. Emphasis is placed on postmitotic tissues, such as neuronal, muscular, and red blood cells, which appear to be those most frequently studied with respect to aging. Additionally, models for the study of aging are discussed in a number of organisms, such as *Caenorhabditis elegans*, senescence-accelerated probe-8 mice (SAMP8), naked mole-rat (*Heterocephalus glaber*), and the beagle canine. Proteomic studies in specific tissues and organisms have revealed the extensive involvement of reactive oxygen species (ROS) and oxidative stress in aging.

## 1. Introduction

Despite the great research efforts performed since molecular techniques have emerged, it is unquestionable that the step-by-step approach of studying one gene or one protein at a time, even if their partners could eventually be unveiled, is a parsimonious endeavor. Therefore, a more integral and holistic approach is required. Within this context, genomic and proteomic studies, mainly by microarray for messenger RNA (mRNA) and two-dimensional (2D) electrophoresis for protein expression profiles, would eventually elicit the comprehension of the entire process of cell function, and also that at tissue and organ levels, which in turn will provide us with a wider panorama and lead us to a more comprehensive understanding of the aging mechanisms and the intrinsic role of reactive oxygen species (ROS) at a molecular level.

Both approaches yield a large amount of information from every group of cells, tissue, or organ condition, both *in vivo* and *in vitro*, under certain circumstances, and at a particular developmental time. In this review, we present a compilation of advances of the influence of oxidative stress during aging, obtained by means of a proteomic approach in different cellular types, tissue types, or animal models. The second major global approach for expression analysis, by gene expression profile by microarrays, will be reviewed elsewhere.

The existence of free radicals, such as chemical entities, was inferred 100 years ago, but their importance in biological systems was not recognized until the mid-1950s; nonetheless, for the majority of the remaining 20th century, ROS were considered “*a type of biochemical rusting agent that caused stochastic tissue damage and disease*” [1]. Now, in the 21st century, reactive oxygen biochemistry is quite relevant among the biomedical sciences, and it is currently recognized that nearly every disease involves some degree of oxidative stress. Additionally, it is recognized that ROS are produced in a well-regulated manner to help maintain homeostasis at the cellular level in normal, healthy tissue [1]; but, when ROS concentrations exceed the cell's antioxidant capacity, severe damage can occur and the organism experiences oxidative stress [2]. The damaging effects of these concentrations have been implicated in aging [3, 4], and also in neurodegenerative diseases such as Alzheimer's disease and Parkinson's disease, and in other chronic and degenerative diseases such as cancer, atherosclerosis, diabetes, and heart disease [5].

As early as 1956, Denham Harman proposed the “Free Radical Theory of Aging” [6]; additionally, other studies from Gilbert, Chance, and Commoner can be considered as the “founders” of reactive oxygen biochemistry. However, by 1980, oxyradicals were accepted biological entities, though their significance for aging and disease remained generally unappreciated [1]. Around 1980, the Stadtman group began to investigate the nature and consequences of protein oxidation *in vitro* and *in vivo*. These authors measured protein carbonyl groups as indices of oxidative damage and applied these techniques to the study of protein oxidation in aging tissue, describing the first, detailed determinations of protein oxidation in models of human aging [7, 8] and giving rise to serious consideration of oxidative stress as a pathological factor.

The application of genomics, proteomics, and even metabolomics to the research on aging, as well as the study of epigenetic influences that are able to induce histone and DNA modifications and influence enzyme activity, would increase our understanding of the origin and development of the different process that contributes to this unavoidable life consequence, senescence. Epigenetic mechanisms are typically associated with the aging process and age-related diseases and may have significant roles to play in the presence of oxidative stress during aging, thereby enabling the establishment of specific diagnostic profiles and therapeutic templates that could aid in improving Quality of Life (QOL) at advanced ages (for a striking revision regarding the relationship between epigenetic factors and aging, see [9]).

## 2. Global Approaches to the Study of Oxidative Stress in Aging at the Molecular Level

**Figure 1:**
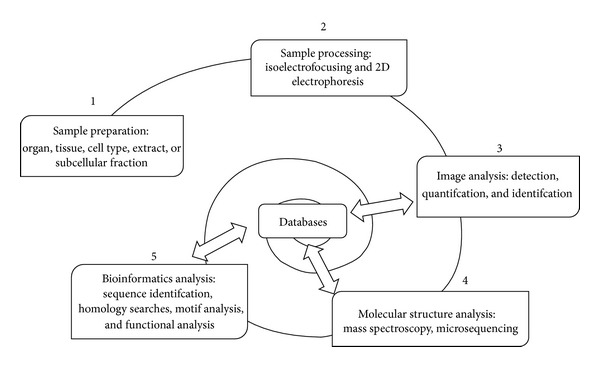
General stages of the proteomic analysis.

One of the major goals of Gerontology is to understand the complex mechanisms involved in aging at the molecular, cellular, and organ levels that would also make the understanding of age-related diseases possible. Because of practical limitations in studying the aging process in humans *in vivo*, animal models are frequently utilized [10]; however, differences in longevity between the majority of experimental animal models and humans make analysis somewhat difficult. Despite these limitations, research in this area has accelerated with the application of high-throughput technologies such as microarrays and mass spectrometry (MS). Once applied, the information generated must be analyzed accurately; thus, the use of bioinformatics is highly relevant [11]. One of the great advantages of the study of the proteomic is that the huge amount of information generated by a particular set of experiments, as occurs in genomics studies also, can be deposited in large databases, which in turn would accelerate, in the near future, experimental work and improve the results obtained, particularly in the study of aging as a comprehensive biological phenomenon (Figure 1). While aging has been considered a stochastic process, widespread opinion at present takes into account the existence of a strictly regulated system that fine-tunes the lifespan of an organism through modulation of its responses to oxidative stress [12].

Research on ROS and oxidative stress has become a rapidly growing and evolving subject of study. Specifically in the field of aging studies, a review of the PubMed database (http://www.ncbi.nlm.nih.gov/) under the terms (“oxidative stress” OR “reactive oxygen species”) AND aging AND microarray or (“oxidative stress” OR “reactive oxygen species”) AND aging AND proteomic (both restricted to title/abstract search fields) shows the increasing interest in these integral molecular approaches during the last decade.

It is relevant to bear in mind the relevance of posttranslational modifications that regulate the activity of proteins inside the cell. Thus, the number of distinct protein functionalities exceeds the number of protein sequences and concentrations; therefore, one must establish the relative concentration, location, and posttranslational modification of each isoform in order to completely characterize protein function.

## 3. Accumulation of Altered Posttranslational Modifications and Lack of Adequate Protein Degradation Are Involved in Aging

During the normal aging process and also in age-related diseases such as atherosclerosis, cataracts, type 2 diabetes, and neurodegenerative diseases, proteins are the targets of several posttranslational deleterious modifications that alter their biological functions (Table 1). ROS increase oxidative stress [13–15] and reactive nitrogen species (RNS) yield nitrosative stress [15]. Together, as well as in conjunction with other toxic compounds, such as dicarbonyl and reactive aldehydes, which are mainly responsible for this damage [16, 17], which in several cases translates into clinical pathology.

**Figure 2:**
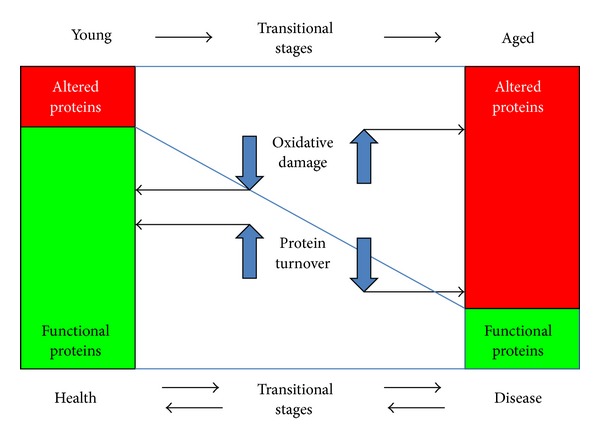
Alteration of proteins by oxidative damage and their turnover. The balance between functional proteins, present in young or healthy organisms, and detrimental or altered proteins, present in a large proportion of aged or diseased organisms, depends mainly on their modification and turnover. If proteins are affected by an increase in oxidative damage or by a low protein turnover, altered proteins accumulate, in contrast to when oxidative damage diminishes and protein turnover increases, when functional proteins increase their proportion and the organism transits to a healthy stage.

Additionally, protein degradation is integral for maintaining a healthy and functional proteome, particularly for turning over misfolded and damaged proteins. Removal of oxidized proteins first involves selective recognition of the modification and afterward, either their repair or their degradation [18]. Protein concentration increases with both decreased degradation and increased synthesis; yet, while decreased degradation results in the accumulation of “old” proteins, increased synthesis does not [19]. Thus, the age-related accumulation of damaged proteins is thought to result from both the increased occurrence of damage, which is due, at least in part, to alterations in the detoxification of the damaging agents, and from the decreased efficiency of the different systems involved in the elimination of damaged proteins [20]. This relationship is illustrated in Figure 2.

Protein quality, but not necessarily quantity, is altered in a disease state and can be reversed by appropriate treatment. This has been demonstrated, for example, in the case of apolipoprotein A1 (ApoA1) in type 1 diabetes, in which ApoA-1 proteins acutely acquired damage during insulin deprivation, providing a feasible mechanism for the association between chronically poor glycemic control and higher levels of protein oxidation in diabetes [21]. In fact, it may be that this rapid aging of ApoA-1, a key protein in lipoprotein metabolism, causes a higher risk of macrovascular disease in persons with type 1 diabetes [22]. Therefore, it has been proposed that aging, as well as certain pathologies such as diabetes, insulin resistance, and metabolic syndrome, are caused in part by the disproportionate accumulation of damaged and dysfunctional proteins, rather than by their increased concentration per se, by the impairing of cellular degradation systems, whereby oxidized proteins that would normally be targeted for degradation accumulate due to age-related slowing of degradation pathways, which are easily overwhelmed by an excess of posttranslational stress [19, 20, 23–28]. Accordingly, observational studies in animals suggest that enhancing protein degradation by caloric restriction and aerobic exercise may retard aging and reverse age-associated pathologies, and it has been hypothesized that caloric restriction helps to modulate the inflammatory process, subsequently leading to the reduction of chronic diseases known to compromise the functional longevity of humans [29]. Notwithstanding this, whether these strategies can promote similar improvements in humans remains to be shown [19].

Oxidation of proteins targets them for degradation; however, extensive oxidation acts against their recycling because it inhibits proteolysis, leading to pathological accumulation and deposition [28, 30]. Modification of proteins also can modify an active site, block a phosphorylation site, or disrupt a binding site for substrates, cofactors, or partner proteins. Furthermore, it can create new epitopes for antibody recognition and induce autoimmune disorders [31, 32].

Although it is widely recognized that cellular aging causes changes in the proteome, the nature and targets of these changes and their consequences have not yet been completely identified. It is noteworthy that accumulative oxidative posttranslational modifications are relevant only if these detected modifications are connected to functional consequences [33]. But in addition, it is relevant to consider that a slight modification in low abundance proteins may be of physiological importance; therefore, many proteomic studies have been undertaken to identify modified proteins. Distinguishing between inconsequential modifications and functionally significant ones requires careful biochemical and biophysical analysis of target proteins. Thus, proteomic approaches represent powerful tools to address these questions by identifying the targeted proteins and the extent of their modifications.

## 4. Oxidation of Proteins Directly Affects Energetic and Metabolic Pathways and Is Related to Longevity

Oxidation-reduction (Redox) regulatory control and oxidative stress are two sides of the same coin: oxidation of proteins can modify proteins under reversible Redox regulatory control or, alternatively, can result in reversible or irreversible oxidative damage. Proteins are very sensitive to the action of ROS [16] and represent nearly 70% of their targeted entities [34]; in addition, ROS can oxidize membrane lipids, generating intermediary compounds that have a longer lifespan than ROS and that can diffuse into the cell, acting as a “toxic second messenger,” amplifying the damage of free radicals [35]. Recently, systemic Redox regulation has been recognized as a highly important element for longevity in a group of Japanese semisuper centenarians (>105 years of age), who probably escape from many serious chronic and age-related diseases by their ability to deal with a variety of stresses, including oxidative stress [36].

Therefore, recent proposals tend to prevent, rather than counteract, ROS production, particularly at the mitochondrial level, during oxidative metabolism (the mitochondrial free radical theory of aging) [37]. Although there is evidence that ROS production in the human skeletal muscle is lower in mitochondria from older subjects [38], adenosine triphosphate (ATP) synthesis was significantly decreased, supporting the concept that aging is associated with a decrease in mitochondrial function, although in this case, ROS production appears to be reduced. In the same study, when older subjects were under a regimen of physical exercise, an improvement was found in their mitochondrial function, as well as a concomitant increase in ROS production [38], which apparently counteracted the effect of aging at the molecular level with respect to mitochondrial function.

Therefore, these apparently contradictory results can be conciliated on the basis of equilibrium between the amount of ROS generated, which can be beneficial or defective, and the amount of oxidized proteins, depending on the tissue and its metabolic status. As a result of the analysis of cumulative evidences, two general characteristics responsible for the degree of high maintenance of long-lived animals emerge: a low generation rate of endogenous damage and the possession of macromolecules that are highly resistant to oxidative modification [37].

To detect protein oxidation, carbonyls are the most commonly employed marker, and the use of 2D gel electrophoresis has provided very useful results for the study of specific carbonylated protein spots during oxidative stress and replicative senescence [39, 40]. Although MS is currently the most versatile technology in proteomics for the identification of proteins and their carbonylated residues, some limitations have led to the development of alternative strategies, such as fluorescent probes, which are able to detect lower abundance carbonylated proteins [20]. Additionally, cysteine oxidation became important because these lie precisely at the interface between Redox-sensitive and oxidative damage by ROS or nitrogen species during stress [41]. Interestingly, a highly significant inverse correlation between long lifespan and the percentage of mitochondrial cysteine is found in metaexaminations of genomic sequences (mitochondrial DNA) from 218 animal species (chordates and arthropods) [42], supporting the previously mentioned free radical theory of aging and point out the relevance of the vulnerability of the proteins in the organism to oxidative stress in terms of its lifespan.

Taking into account the alterations of metabolism in humans, the Cornelia de Lange syndrome is a rare multisystem disorder characterized by distinctive craniofacial dysmorphia, upper limb malformations, hirsutism, microcephaly, cardiac defects, gastroesophageal dysfunction, growth retardation, and neurodevelopment impairment ranging from moderate to severe, with a wide range of variability [43]. Patients present a premature aging process, and it is likely that a reduction in energy and the downregulation of proteins involved in antioxidant and detoxification pathways could lead to premature physiological aging and genome instability [44].

On the other hand, a possible model of longevity has been described; it is based on the knocking out of type 5 adenylyl cyclase (AC5) [45]. Adenylyl cyclase (AC) is a key enzyme that catalyzes the synthesis of cyclic adenosine monophosphate (cAMP) from ATP and it plays a pivotal role in *β*-adrenergic receptor signaling. In these AC5 KO mice, activation of the Raf/Mitogen-activated protein kinase/MAP kinase kinase (Raf/MEK/ERK) signaling pathway is present, which in turn promotes the upregulation of Mn-superoxide dismutase (Mn-SOD) and results in protection from oxidative stress and apoptosis, retarding aging phenotypes in the heart and bone and increasing resistance to stress, which leads to longevity in the lifespan, suggesting that retarding aging in an individual organ could be a fundamental therapy to prevent age-related diseases [46].

Delving deeper into the identification of proteins involved or affected by aging, postmitotic tissues have received more attention. Neurons, myocytes, and red blood cells have been studied during the aging process, and certain particular and some common factors have been discovered. In the following sections, the main advances in proteomics studies of these post-mitotic cells are presented.

## 5. Proteomic Studies Further Support Parallelism between Aging and Neurodegenerative Diseases

An increase in oxidative stress in the brain is part of normal aging and is related directly to decreased neurological activities and inversely to lifespan [47]. Common pathological pathways that are implicated both in aging and in the development of neurodegenerative disease include free radical damage and decreased energy production as characteristic hallmarks [48]. Consistent with this idea, proteins that increase with aging in the mice hippocampus are mainly enzymes that mediate energy production and oxidative stress [49]. In addition, one of the cellular processes that are altered mainly in the aging hippocampus is that of oxidative stress, as well as that of protein processing [50].

Consistent with previous ideas, wide proteomic analysis of brains during aging in mice identifies 40 proteins that exhibit changes in their natural pattern during the mouse lifespan (from 4 days to 15 months of age), showing that six proteins increased and 27 decreased in various ways. When analyzed together, the biological processes in which those proteins are involved correspond mainly to the following: protein metabolic processes (more than one third); transport (one third); nucleotide and nucleic acid metabolic process (one fourth); intracellular signal cascade (nearly one fifth), and response to stress proteins (more than one sixth). Additionally, about one fifth of the identified proteins can be localized in the mitochondria and the majority of these are related to energy metabolism [51], providing a broad panorama of proteomic changes in the brain during aging.

In addition, there is increasing evidence that protein oxidation is involved in the pathogenesis of Alzheimer's disease (AD), a neurodegenerative disorder associated with cognitive decline, oxidative stress, and aging [52–56]. Two initial studies using the proteomic approach have identified proteins that are specifically oxidized in AD [54, 55]. In the first study, three key enzymes in cellular metabolism, creatine kinase BB (CK BB), glutamine synthase (GS), and ubiquitin carboxy-terminal hydrolase L-1 (UCH L1), resulted as specific targets of protein oxidation in the brain of AD patients. In the second, a couple of additional targets were detected: dihydropyridine-related protein-2 (DRP-2), involved in axonal growth, and *α*-enolase, involved in glycolysis for energy metabolism and therefore related with the cerebral decrease of energy metabolism. These results strongly suggest that the process of free radical-mediated protein modification may be a crucial event in AD and that protein oxidation is a relevant part of the mechanism of neurodegeneration in AD brain.

Although in a pilot study of abundant carbonylated proteins in the cerebrospinal fluid of probable AD patients the extent of carbonylation did not vary in general, two proteins were detected that were highly carbonylated when compared to controls: immunoglobulin *λ* light chains and one unidentified protein [57], which suggests that further studies could be performed focusing on less abundant proteins, modified by oxidative stress, to detect some probable markers of early pathological stages. Therefore, the establishment of differences in protein oxidation state may provide a diagnostic tool for neurodegenerative diseases.

Additionally, Weinreb et al. found a significant parallelism in the protein profile affected between aging and neurodegenerative diseases in the hippocampus of rats [58]. They found that in the aged hippocampus, oxidative stress and mitochondrial dysfunction are important and that in treatment with an anti-AD drug, Ladostigil, or with an anti-Parkinson drug, Rasagiline, both drugs reversed the effect of aging on various mitochondrial and key regulator genes involved in neurodegeneration, cell survival, synaptogenesis, oxidation, and metabolism. Another consequence of oxidative stress in the brain includes the generation of RNS. The cerebellum is especially vulnerable to oxidative stress and exhibits an age-dependent increase of total 3-nitrotyrosine (3-NT) [59, 60], and some proteins have been identified as targets for nitration [59].

Additionally, in cultured neurons exposed to amyloid beta (A*β*) (1–42), two proteins are significantly oxidized: 14-3-3*ξ* and glyceraldehyde-3-phosphate dehydrogenase (GAPDH), and pretreatment with *γ*-glutamylcysteine ethyl ester, a compound that supplies the limiting substrate for antioxidant glutathione synthesis, protects both proteins from oxidation by A*β* 1–42 [61], which is consistent with the notion that antioxidant therapies may potentially be effective in slowing or ameliorating the neurodegenerative disease process [56, 62].

Conversely, the other cell type that is more abundant in the central nervous system, the glial cells, are more resistant than neurons to oxidative stress and are able to respond to protect them [63–66]. Surprisingly, proteomic studies that focus on the role of glial cells during aging, or in neurodegenerative diseases, in response to oxidative stress are very scarce. Miura et al. proposed that aging does not suppress the astrocytic capability to respond to oxidative stress. The authors found that *α*-tubulin was subjected to tyrosyl phosphorylation by H_2_O_2_-exposure and that aging enhanced this phosphorylation and prevented the formation of microtubules, but aging does not suppress the responses aimed at cell protection against severe oxidative stress [67]. Therefore, proteomic studies on the response of glial cells to oxidative stress during aging and in neurodegenerative diseases, focusing on their helping role for neuron metabolism, represent a promising avenue that is yet to be explored.

## 6. Aging in Cardiac and Skeletal Muscles Altered Energy Metabolism and Mitochondria

Cardiac performance declines with age [68] in a clear association with oxidative stress [3]. Accordingly, aging is a factor for cardiovascular disease. It appears that the protein signature or proteomic phenotype that changes during aging in the rodent heart renders the tissue more sensitive to oxidative stress damage with age by modifying the energy metabolism, particularly carbohydrate metabolism, fatty acid oxidation, cellular respiration, and energy production and their capacity to respond to oxidative stress [69].

In the aged rat heart, several proteins have been identified as differentially expressed when compared with those of young hearts [69–71], although there are many differences among studies, probably derived from methodologically different approaches and species particularities (mouse or rat), also due to the fact that many proteins change consistently during aging. Additionally, a study of a heart failure model, transverse aortic constriction in mice, demonstrated a differentially expressed protein expression of structural, signaling, and redox proteins [72]. Thus, it is possible to establish parallelism between aging heart dynamics and heart failure, both altering structural proteins as well as the oxidative metabolic profile and affecting the heart's capacity to respond adequately to oxidative stress during aging or under pathological distress.

Among the functional consequences of oxidative stress induced by ROS and RNS on cardiac and skeletal muscle tissue during aging, we find protein nitration [73], which may affect protein structure, function, and turnover. Thus, the accumulation of nitrated proteins in cardiac and skeletal muscle tissue [74, 75] may define the progress of biological aging or of any pathology.

## 7. During Red Blood Cell Aging, Hemoglobin-Generated Oxidants That Affect Cellular Membrane and Cytoskeleton

Many of the cell processes associated with the physiological removal of red blood cells (RBC) involve oxidative stress, which is generated by both endogenous hemoglobin (Hb) auto-oxidation and exogenous oxidants, which can result in functional impairment and in cellular aging [76]. The predominant factor that determines oxidative stress in RBC is Hb. The superoxide, H_2_O_2_, hydroxyl radicals, ferrylHb, oxoferrylHb, and peroxynitrite generated by redox reactions near the membrane can damage RBC membrane proteins, lipids, and the cytoskeleton, which are responsible for maintaining the RBC shape and deformability, thus being able to damage and promote cellular aging [77]. Consequently, instead of required large concentrations of antioxidants to neutralize the ROS species formed, blocking Hb interaction with the membrane will make it possible to eliminate Hb-generated oxidants, preventing oxidative stress in RBC [76]. In RBC, and due to their extensive use for blood transfusions, their oxidative stress is also highly relevant during long-term storage [78]. Consequently, the proteomic approach has been useful to identify molecular markers, such as Prx2, as a candidate biomarker for RBC oxidative injuries under blood bank conditions [79], possibly to be utilized in future blood component programs to improve the quality of stored RBC and to limit or avoid the risk of posttransfusional complications.

**Figure 3:**
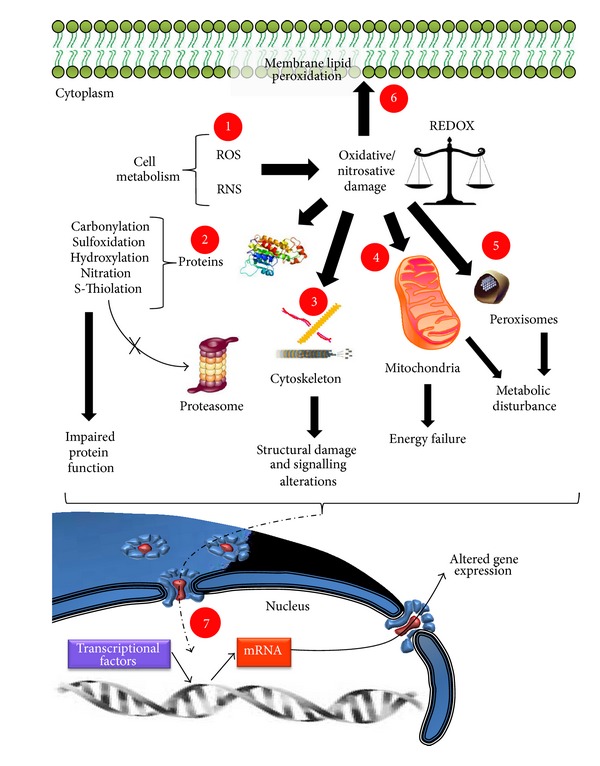
Cellular dysfunctions in aging or in age-related diseases by oxidative stress imbalance. (1) Cell metabolism generates reactive oxygen species (ROS) and reactive nitrogen species (RNS), which in turn causes oxidative/nitrosative damage. (2) Proteins are the most affected macromolecules by oxidative stress, undergoing several modifications that avoid their being correctly degraded and recycled by the proteasome, thus generating impaired protein function. (3) Oxidative stress also directly affects cytoskeletal proteins, causing structural damage and signaling alterations. (4) On affecting the mitochondria, oxidative stress alters energy production and (5) on affecting peroxisomes, oxidative stress alters correct metabolic functioning. (6) Oxidative stress also affects the cellular membrane. (7) Finally, all of the previously mentioned affections cause an alteration in the transcriptional activity of the cell, leading to an altered gene expression that in turn leads the cell to the aging process or to degenerative disease.

In brief, and beyond the cell type affected, on the basis of extensive experimental results, the main cellular dysfunctions directly caused by oxidative stress imbalance and by uncontrolled generation of ROS can be summarized as follows (Figure 3). Whether from self-cell metabolism or from extracellular sources generating an uncontrolled increase of ROS and RNS, both of these produce cell damage by modifying proteins (mainly by carbonylation, sulfoxidation, hydroxylation, nitration, and S-thiolation) and avoiding the recycling of these by the proteasome, yielding impaired protein function. Additionally, oxidative stress can directly affect the cellular membrane, causing lipid peroxidation, thus instability, and can also modify cytoskeletal proteins, causing structural damage and moreover altering signaling pathways. Furthermore, oxidative stress directly affects mitochondria and peroxisomes, thereby altering cell metabolism and energy production. Taken together, these alterations would be able, in some manner, to distress nuclear transcription and generate altered gene expression during normal aging, or leading to disease. Although this is a general panorama, there are more specific alterations depending on the cellular type affected or on the tissue or organ altered. Therefore, it is a priority to establish more models to study the whole effect of oxidative stress during aging and disease.

## 8. Particular Organisms as Alternative Models for Studying the Proteomics of Oxidative Stress during Aging

In addition to the more widely used models employed to study aging (such as rodents like mice or rats, cultured line cells, or even tissue fragments, blood, or serum), other particular organisms represent suitable alternatives for approaching the problem. In Table 2, we summarize some of the main works that, based on their wide proteomic approach, deal with the aging process, selecting those that have obtained results that are clearly related with the involvement of oxidative stress and ROS in the aging process. The following is a brief summary of some particular models and their contribution to elucidating the participation of oxidative stress during aging.

The invertebrate *Caenorhabditis elegans* has been successfully used to study the aging process. It has been described that some proteins are involved in both cellular senescence and the ROS-induced condition. By using interfering RNA (iRNA) against some genes, a substantial reduction can be caused in adult lifespan, and the defensive mechanism against external oxidative stress is also disturbed [80]. Therefore, some proteins whose expression is increased with cellular senescence and oxidative stress play a protective role against these processes. Additionally, when *C. elegans* is submitted to oxidative stress through sublethal short-treatment of peroxide (H_2_O_2_) stress, the majority of worms experience severe, yet fully reversible, behavioral changes that are highly reminiscent of well-known age-related changes [83], such as declines in body movement, pharyngeal pumping, and reproduction, as well as morphological changes and reduced metabolic activity [83], which supports the Harman free radical theory of aging, but would also include the potentially beneficial aspects of ROS as modulatory second messengers that affect stress resistance and longevity early in life [83]. In another example, *C. elegans xpa-1* mutants, which are ultraviolet-light (UV)-sensitive and that have reduced capacity to repair UV-induced DNA damage [112], exhibit oxidative stress and its antioxidant defenses are induced, and they also show polyubiquitinated protein accumulation [84]. Obviously, there are differences between nematode (invertebrate) and mammalian (vertebrate) systems, but the fundamental mechanism of cellular senescence may be evolutionarily conserved.

Another very attractive model for studying the effect of oxidative stress during aging is the senescence-accelerated probe-8 (SAMP8) mouse, which exhibits age-related deterioration in memory and learning, along with an increase in oxidative markers and which is considered a useful model for the study of AD [113]. In AD, it has been demonstrated that treatment with *α*-lipoic acid, a coenzyme involved in the production of ATP in mitochondria and a potent antioxidant [114, 115], is able to reduce oxidative modification and increase the protein level of *α*-enolase, suggesting the possibility that the reduced glucose metabolism and neurochemical alterations in SAMP8 mouse brains can be reversed [116]. Additionally, it has been demonstrated that carbonyl modification of Cu, Zn-superoxide dismutase (Cu, Zn-SOD) in liver and hippocampal cholinergic neurostimulating peptide-precursor protein (HCNP-pp) in the brain were higher in SAMP8 compared with the control, SAMR1. Therefore, progressive accumulation of oxidative damage to Cu, Zn-SOD may cause dysfunction of the defense systems against oxidative stress in SAMP8 with higher oxidative states, leading to the acceleration of aging [85].

A different and very interesting organism for studying the effect of oxidative stress during aging is the naked mole-rat (*H. glaber*) because it has very low metabolic and respiratory rates and its protein structure and function is not apparently affected by either oxidative stress or carbonylation during aging, probably due to a particular characteristic of the cellular environment that maintains the functional structure of proteins [104]. As an example, activation of the Nuclear factor [(erythroid-derived 2)-like 2] (Nrf2) antioxidant response pathway, which increases the transcription of antioxidant response-element genes, proteasomes, and antioxidants and which affects the efficient maintenance of protein homeostasis, can protect proteins from misfolding or aggregation by oxidative stress [117, 118], being part of a protective cellular environment that efficiently maintains protein homeostasis as part of a potential plausible mechanism that explains the exceptional longevity of the naked mole-rat [104].

Finally, an attractive model for the aging human brain is the aging beagle (canine) brain, especially also as a model of AD [119–122]. In the aging canine brain, a proteomics study reveals that a combined treatment of antioxidant-fortified food and an enriched environment reduces the levels of oxidative damage, improves the antioxidant reserve systems, increases the activity and expression of key endogenous antioxidant enzymes, and may contribute to improvements in learning and memory [106].

## 9. Concluding Remarks

One limitation of some of the reports presented here is that details regarding animal ages, care, and behavior assessments/measures are limited, which impedes cross-study comparison and meta-analyses. Also, cell types and their particular characteristics rendered comparison of the effect of ROS on RBC or neurons difficult, for example, as well as under *in vitro* or *in vivo* conditions. Thus, therefore more and wider studies are needed.

In humans, it is difficult to compare among proteomic studies because of insufficient characterization of the study material, the small number of patients involved in studies, and variations in experimental designs. At present, basic aging research has arrived at a pharmaceutical phase, with the testing of novel drugs designed to extend a healthy life by targeting specific biochemical pathways, perhaps in specific organs [123]. In this respect, the National Institute on Aging Interventions Testing Program (ITP) experimentally evaluates chemical compounds with potential senescence-retarding effects that can be administered to mice in food or water [124]. While initial results are far from surprising, the experimental design is robust; therefore, it will be useful in order to develop a similar program in mouse genetics in aging.

It is evident that the sole fact of identifying the whole genome sequence of an organism, or to know the whole isoforms and modifications of its products (proteins), is not sufficient for complete elucidation of the aging process. It is necessary to integrate all of this information in a functional manner that reflects more precisely the real situation. Therefore, as important as the generation of all “omic” information is, the development of instruments to analyze and evaluate this efficiently is equally important. In this regard, bioinformatics and computational biology are devoted to performing these analyses, both based on systems biology, that is, the construction of gene, protein, and metabolic pathway networks that interact among them to constitute functional modules (Figure 1). In turn, they integrate design models for prediction from clinical phenotypes to diagnostic and therapeutic strategies after experimentation takes place. Albeit proteomics has already contributed relevant insights in the field of aging research and attempts have been made, in animal models such as mice to map aging-related brain proteins within the context of the biological processes involved [51]; a reference mapping of proteins in healthy aging human subjects has yet to be performed. Nonetheless, with the continued advances in proteomic technology, the study of the proteome during aging is entering a brand new phase of discovery.

## Figures and Tables

**Table 1:** Major posttranslational protein oxidative modifications during oxidative stress mediated aging.

Type of modification	Consists of
Irreversible	
Carbonylation	Covalent adduction of lipid aldehydes, often six, nine, or 12 carbons, to the side chains of lysine, histidine, and cysteine residues
3-Nitrotyrosilation	Formed between reactive nitrogen species and a protein's tyrosine residue
Reversible	
S-Sulfenation	Generation of sulfur-hydroxylation product (P-SOH) may be a prelude to sulfination, sulfonation, disulfide bond formation, and sulfenyl-amide bond formation
S-Nitrosylation	Covalent incorporation of a nitric oxide moiety into thiol groups to form S-nitrosothiol (SNO)
S-Glutathionylation	Covalent attachment of glutathione (GSH) to protein thiol groups
Disulfide formation	Disulfide bonds are usually formed from the oxidation of sulfhydryl (–SH) groups
4-Hydroxy-2-nonenal (HNE) modification	Is a major lipid peroxidation product formed during oxidative stress

**Table 2:** Comprehensive summary of proteomic studies focused on aging that involves oxidative stress-related proteins.

Animal model (specie) and tissue	Sample and age	Results related to oxidative stress or ROS influence on aging	Main proteins altered in aging*	References
Invertebrate *Caenorhabditis elegans* (worm)	*In vitro* knocked down by RNAi	Ten iRNA tested caused substantial reduction in adult lifespan. When these genes are disturbed defensive mechanisms against oxidative stress become altered.	UBH-1, UBH-3, PRDX2, PRDX3, AMPK-*β*1, AMPK-*β*2, LBP-4, LBP-6, LBP-9, RHI-1	[80]
	*In vitro* knocked down by RNAi for K10C2.4	K10C2.4 RNAi activates oxidative stress and endoplasmic reticulum stress response in the worm intestine by accumulation of tyrosine metabolites.	Enzymes in the tyrosine degradation pathway	[81]
	Long-lived *daf-2*(e1370) strain	Components of the enhanced longevity system identified in *daf-2 *deficient mutant include the alpha-crystallin family of small heat shock proteins, anti-ROS defense systems, and cellular phase II detoxification. GSTP were significantly URg, which detoxify and/or bind short-chain aldehydic natural toxic products of lipid peroxidation and long-chained fatty-acids at physiologically relevant concentrations, indicating a role in longevity.	GPX, SOD1, NME, RPS12, STK, LBP-6, HSP-12.6, HSP-12.3	[82]
	Exposure of prdx-2 defective worms under H_2_O_2_-induced OS	Identified oxidation-sensitive cysteins in 40 different proteins involved in mobility (muscle contraction), feeding, protein translation, homeostasis, and ATP regeneration.	MYO-2, LET-75, EFT-1, HSP1, NME	[83]
	*xpa-1 *mutant UV-sensitive with shortened lifespan	Proteome changes in xpa-1 mutants correspond to transcriptome modulation by suffering oxidative stress and inducing antioxidative defenses. Polyubiquinated proteins accumulate, cyclopurine levels are reduced, and lesion-detection enzymes play active roles to generate a genomic stress signal.	NTH-1, XPC-1, DDB-1	[84]

Rodents *Mus musculus* (mice)	KO mice for 5 adenylyl cyclase (AC5)	AC5 KO mice are protected from aging-induced cardiomyopathy and their fibroblasts exhibited ERK-dependent resistance to oxidative stress. AC5 KO leads to upregulation of the Raf/MEK/ERK signaling pathway, which in turn mediates upregulation of SOD, an important mechanism mediating lifespan extension and stress resistance.	Increased: RSK, p-Bad, Bcl-xl, XIAP, HSP70, p-ERK, p-Raf-1	[46]
SAMP8	Brain and liver from SAMP8	Progressive accumulation of oxidative damage to Cu, Zn-SOD may cause a dysfunction of defense systems against oxidative stress in SAMP8, with a higher oxidative stress and leading to the acceleration of aging.	SOD1, HCNP-pp	[85]
	Hippocampus and cortex from 5 to 15 month old SAMP8	7 protein are related to age rather than strain and might be associated with brain aging process. One protein might be specifically associated with pathologically accelerated aging in SAMP8 mice; HEBP1.	NDRG2, enolase 2, SOD1, myosin, two unnamed protein (gi∣74214304; gi∣74178239), HEBP1	[86]
Brain	Brain tissue from 3 weeks to 18 months old C57B mice	Carbonylated proteins increased with aging are involved in cytoskeletal organization, mitochondrial energy metabolism, redox regulation (oxidative damage), and signal transduction.	Approximately 100 carbonylated proteins	[87]
	Brain tissue from 3-, 6-, 12- to 15-month-old male Kunming mice	60 proteins vary their expression on aging; 27 of them decrease, may be responsible for brain aging. Related with decline of protein quality control, shortage of energy and reducing agent, increase of DNA damage and transcription detuning, and disturbance of synaptic transport and ion signals. 6 proteins increase, may be involved in antiaging processes.	PSMA6, PSMA3, CALR, UCHL3, VCP, GLUD1, IDH1, UQCRC2, UBE2N, CALB1, HNRPA2/B1, AMPH, TKT, CKMT1, MRPL37, TPI1	[49]
Brain	Adult NPCs from brain of C57BL/6 mice from 3, 15–18 months of age	Aging is correlated with a loss of mitochondria and oxidative metabolism in NPCs. A coordinated shift in protein expression, subcellular structure, and metabolic physiology in aging NPCs, allowing resistance to hypoxia and mitochondrial inhibition. 124 proteins result as age-related.	Increased: PGK1, SEPT9. Decreased: ATP5 *α* and *β*	[88]
Liver	Livers from male C57BL6/J mice of 10-week-old and 18-month-old. Peroxisome enriched fraction	Most of the proteins identified are related to ROS production/breakdown; however, high biological variability between individuals is even more pronounced than changes induced by aging.	EPHX2, Acaa1, Pipox, Amy2a, Decr2, Phb2, COX6c, UQCRC2	[89]
Kidney	Male and female mice CD1-Swiss outbred strain of 28, 52, 76-week-old	Differential protein expression of 8 aging related proteins (both genders). Increase in oxidative and proteolytic proteins and decrease in glycolytic proteins, and antioxidant enzymes with aging.	ATP syntase, Transferrin, HSP9A, Hibadh, IDH1	[90]
Cardiac muscle	Hearts from male CB6F1 mice from 3, 15 to 23 months old	Detected age-related alterations in the levels of 73 proteins. Mithocondrial metabolism is affected and a net loss in antioxidants occurs with aging.	Mortalin, PRDX3, EPHX, SOD1, SOD2	[70]
Adipose tissue	Male mutant mice deficient in Zmpste24 metalloproteinase	Zmpste24 deficiency causes premature aging. It enhanced lipolysis, fatty acid biogenesis, and *β*-oxidation as well as decreased fatty acid reesterification. Also URg protein networks related to tricarboxylic acid cycle and oxidative phosphorylation and increased mitochondrial response to oxidative stress and cytoskeleton. 37 proteins URg and 9 DRg.	Increased: ME1, PRDX3, HMGB1, CPT1, UCP1; Decreased: PCK1, vimentin isoforms	[91]
Macrophages	Peritoneal macrophages from male Balb/c mice (3-4 and 14-15 months)	An age-dependent increase in the extent of recruitment of macrophages into the peritoneum, as well as *ex vivo * functional changes involving enhanced nitric oxide production under resting conditions. Identified age-dependent increases in levels of proteins linked to immune cell pathways under basal conditions and following LPS activation. Immune pathways URg in macrophages isolated from aged mice include proteins critical to the formation of the immunoproteasome.	Hundreds of proteins	[92]

*Rattus novergicus *(rat)	Male Wistar rats weighing 80–90 g, 6 and 24 months old	A beneficial role for virgin olive oil in modulating inflammation, homeostasis, oxidative stress, and cardiovascular risk during aging. Diet diminishes in general the changes that occurred with age.	Decreased: HPX, HP, AHSG, PRDX2, FGg, T-KNG, APO H, APO E, APO A-IV Increased: APO A-1	[93]
	Serum from young and old Fischer 344 rats	16 of the modified proteins by peroxynitrite and 4-hydroxy-2-nonenal are involved in blood coagulation, lipid transport, blood pressure regulation, and protease inhibition.	16 modified proteins	[94]
Brain	Hippocampus from 8 to 27-month-old Wistar rats, and also treated with the anti-Parkinson drug; rasagiline or the anti-Alzheimer's disease drug; ladostigil	Significant molecular changes related to neurodegeneration were identified in aged rat hippocampus. Both drugs reversed the effect of aging on the expression of various mitochondrial and key regulator genes involved in neurodegeneration, cell survival, synaptogenesis, oxidation, and metabolism. Changes in proteins related to the iron-mediated oxidative stress pathway, including reduction in antioxidant enzymes. Oxidative stress and mitochondrial dysfunction may play a pivotal role in aging and age-associated neurodegenerative diseases.	Aprox. 200 proteins showed differential expression. NEFL, FTH1, TUFM, PEA15, PEBP, PFN1, CCT2, IDH3A, COX5A, COX5B, PRDX2	[58]
	cerebellum from Fisher 344/Brown Norway rats from 5-, 22- and 34-month-old rats	Genes encoding proteins of stress response and inflammatory processes show a significantly higher age dependent upregulation in the cerebellum suggesting higher levels of oxidative stress. Identification of nitrated proteins.	Ryr3, Lrp2, Nrap, Cnp	[95]
Brain	Brain from male Wistar rats from 12 to 28 months old (hippocampus, cortex, striatum, and cerebellum)	Senescent animals showed significantly higher levels of oxidation. 11 proteins carbonylated in hippocampus, 15 in cortex, 10 in striatum, 11 in cerebellum, associated with significant changes in both cytosolic and mitochondrial redox status in all brain regions analyzed.	Decreased: PK, ATP5a1, ALDOC, CKB, a-enolase. Activity of PK and GAPDH diminished	[48]
	Hypotalamus and hypophysis from male Wistar rats from 3, 12 to 24 months old treated with an antioxidant	Alterations of eEF-2 levels, secondary to lipid peroxidation and adduct formation with aldehydes could contribute to the suboptimal hormone production from these tissues during aging	eEF-2, ALDOA, GSTA, CKB, PPIA, PK, GADPH, INA, CFL1	[96]
	MSC cultures from the tibial and femoral BM of 83-week- and 12-month-old Sprague-Dawley rats	Number of MSCs is reduced in aged animals. Aged MSCs are more susceptible toward senescence and display a lower migratory capacity. Aging affects MSCs antioxidant defense and cytoskeleton turnover.	Several proteins as members of the actin-binding protein family of calponins, galectin-3	[97]
Retina	Fisher 344/Brown Norway F1 rats from 3-4 to 24-25 months old	Decrease of antioxidant enzymes was detected in the old F344BN retina sections and increased presence of ROS and oxidative stress.	Increased: CD46, GABA2, DJ-1, EBP50, Ezrin, Cathepsin D. Decreased: NG, DDAH1, DPPX	[98]
	Primary cell cultures from retinas of newborn (PD 1 or 2) Sprague-Dawley rats under H_2_O_2_-induced OS	Retinal pigmentary epithelium (RPE) and retina have higher O_2_ tension and ROS concentration with aging; this environment may contribute to the pathogenesis and progression of eye diseases. Decreased prohibitin in H_2_O_2_ treated RPE cells may indicate an antioxidative role.	Prohibitin	[99]
Adipose tissue	White adipose tissue from male Wistar rats from 6 and 24-month-old under caloric restriction (CR)	Caloric restriction (CR) improves oxidative stress and prevents age-associated changes in several antioxidant enzymes. Metabolic enzymes involved in energy metabolism and transduction (glucose and lipid), oxidative stress response, cytoskeleton, and iron homeostasis were also modulated by age and/or CR. Several enzymes involved in cell protection against oxidative stress are increased by CR, whereas these protein levels decrease or do not change with age.	133 differentially expressed spots, 57 of which were identified	[100]
Skeletal muscle	Skeletal muscles from Fisher 344/Brown Norway F1 rats, 34 months old	11 nitrated proteins were identified as age-related.	CKM, TPM1, GAPDH, MYL2, ALDOA, PKM, PYGM, NOTCH1, ACTN1, ACTC1, RYR3	[74]
	Gastrocnemius muscle from Lou/c/jall male rats from 7, 18, to 30 months old	Aging is associated with differential expression of myofibrillar regulatory proteins, up-regulation of cytoskeletal proteins, perturbations in the energy metabolism, and detoxification of cytotoxic products.	40 proteins differentially expressed	[101]
	Gastrocnemius muscle of 26-month-old Wistar rats	Mitochondria-enriched fraction revealed an age-related change in 39 protein species. An age-related increase in mitochondrial enzyme activity belonging to the inner membrane system, matrix, outer membrane, and intermembrane space, increasing aerobic-oxidative metabolism, involved in oxidative phosphorylation, ATP formation, and fatty acid oxidation.	Increased: NADH-DH, Immt mitofilin, PRDX3, F1-ATPase, SDH, Fis1, SUCLA2, ACAD, porin VDAC2, UQCRC1, prohibitin	[102]
Cardiac muscle	Left ventricle from Fisher 344 rats from 4 to 24-month-old	117 proteins differentially expressed: 23 signalling proteins, 25 metabolic proteins, 7 fatty acid metabolism, 19 energy metabolism, 13 oxidative stress related (antioxidant proteins and chaperones). First network describing proteins affecting cellular organization and morphology is presented.	*αβ*-Crystallin, GST *π* isoform, GST *μ* type, GST Ω1, C1qbp, HSP90b1, GPX1, DJ-1, SOD2, PRDX5, PRDX3, HSP8	[69]
	Heart from Fisher 344/Brown Norway F1 rats, 5 and 26 months old	48 differentially nitrated proteins were identified that undergo an age-dependent protein tyrosine nitration.	*α*-Enolase, Aldolase, Desmin, ACO1, Aldh6a1, Acaa1a, GAPDH, MDH1, CKM, ETF, SOD2, F1-ATPase, VDAC	[103]
	Heart from Fisher 344/Brown Norway F1 rats, 5 and 34 months old	Abundance of 10 nitrated proteins identified in cardiac tissue increase with age.	N-RAP, neurofibromin, tropomyosin, MYO-HC	[73]
*Heterocephalus glaber *(Naked mole-rat)	Liver, heart, and kidney tissues from naked mole-rats (NMRs; 2 years) and wild-type C57BL/6 mice (0.3 year)	Global protein carbonylation in citosolic fraction was elevated in all three tissues. NMRs have a protective cellular environment which restores enzyme function and prevents formation of oligomers during oxidative stress, modulating structure and function of structural proteins and enzymes. Activation of NRF2 pathway, which increases the transcription of antioxidant response elements, proteasome, antioxidants, and autophagy, could be a potential mechanism for these processes.	TPI, PRDX1	[104]

Porcine *(Sus scrofa) *	Porcine oocyte and effect of caffeine	38 proteins were identified, 23 URg and 3 DRg by aging. Involved in metabolism, stress response, ROS, and cell cycle regulation.	CDK5, PCNA, AHCY, SLC25A6	[105]

Canine *(Canis domesticus) *	Brain from beagle dogs from 8.05 to 12.35 years old. Feeded with antioxidant-fortified food and growth in an enriched environment	Combined treatment (food and environment) significantly decreases protein cabonylation, nitrosylation, and lipid peroxidation, reducing the levels of oxidative damage and improving the antioxidant reserve systems in the brain. Propose a diagram of a functional interacteome of all parietal cortex proteins identified to be significantly less oxidatively modified following the combined treatment.	Decreased: GLUD1, GAPDH, a-Enolase, GST, FSCN1, NF-L. Increased: SOD1, ALDOC, CKB, GLUD1 (P), GAPDH (P)	[106]

Primate *(Homo sapiens) *	Human female of 20–39, 100, and 106–109 years old	Results suggest that systemic redox regulation is important for the longevity of supercentenarians in humans.	Decreased: PON1, APO E. Increased: Hp-b, AMBP, CLU	[36]
Brain	Inferior parietal lobule tissue samples from AD patients autopsy	Protein modification by ROS occurs to a greater extent in AD suggesting a possible role for oxidation-related decrease in protein function in the process of neurodegeneration. Oxidative damage to proteins, assessed by measuring the protein carbonyl content, is involved in several events such as loss in specific protein function, abnormal protein clearance, depletion of the cellular redox-balance and interference with the cell cycle, and, ultimately, neuronal death.	Increased in AD: DRP-2, *α*-Enolase, HSC-71	[54, 55]
CSF	Lumbar CSF samples from probable AD patients	Decreased concentrations of proteins in CSF may also be a secondary event to increased oxidative stress, since excessive carbonylation leads to an enhanced aggregation of proteins. Extent of protein carbonylation can vary between men and women, emphasizing the importance of sex-matched patients when studying carbonylation.	Decreased in AD: PTGDS, IgL, TTR. Increased carbonylation in AD: IgL and one unidentified protein	[57]
Blood	Whole blood from healthy volunteer donors. Stored for various periods	A progressive linkage of typical cytosolic proteins to the membrane was detected, including both antioxidant and metabolic enzymes. This phenomenon was unequivocally related to oxidative stress, since storage under anaerobic conditions suppresses it.	Prx2	[79]
Skin	Fresh punch biopsies from the forearm of 21–30 and 75–92 years old donors	22 proteins were consistently deregulated. Support that aging is linked with increased oxidative stress that could lead to apoptosis* in vivo*.	Mx-A, SOD1, WARS, PIK3r2, proteasomal PA28-*α* and SSP 0107	[107]
Fibroblast	WI-38 human embryonic fibroblasts. Two stages PD < 25 and PD > 42	Oxidized proteins accumulate with aging *in vivo* and during replicative senescence* in vitro*. 37 proteins were modified related to protein quality control, energy metabolism, and cytoskeleton. Impairment of glyoxal- and aldehyde-detoxification mitochondrial systems.	Decrease activity of proteasomal CT-L, PGPH and detoxification GLO1	[39]
	HCA3 human dermal fibroblasts under H_2_O_2_-induced OS	H_2_O_2_-induced senescent like human diploid fibroblasts increase the production of IGFBP-6 protein.	Increased: Collagen 1(VI), collagen 2(I), fibronectin, lumican, MMP-2, IGFBP-6	[108]
	HCA3 human dermal fibroblasts under H_2_O_2_-induced OS	H_2_O_2_ treatment caused elevated levels of TXNRD1. Differences between mRNA versus proteins that vary under oxidative stress may be related to the regulatory mechanism of protein translation under oxidative stress.	Increased: TXNRD1, MMP-3, AURKA Decreased: Akap12, MDH1	[109]
Colon epithelial	Human normal colonic epithelial tissue from 25–30 to 60–65 years old	35 differentially expressed proteins, 16 URg and 19 DRg. Involved in metabolism, energy generation, chaperone, antioxidation, signal transduction, protein folding, and apoptosis.	Increased: ATPB, ETFA, catalase, GPX1, annexin A2, HSP7C; decreased: FUBP1, NDK B, ERp6C, VDAC-2	[110]
MSCs	Human BM-derived MSCs	Differentially expressed proteins under the low glucose condition may provide further information on the aging and differentiation of stem cells.	Increased: ALDH, neuropolypeptide h3, P4HA; Decreased: laminin-BP, actin, Sec 13, RPS12, PSMA1, SOD1, SNAP	[111]

*Proteins based on their human homologue. ROS: reactive oxygen species, URg: upregulated, DRg: downregulated, PD: postnatal day, SAMP8: senescence-accelerated mouse prone 8, NPCs: neural precursor cells, MSCs: mesenquimal stem cells, BM: bone marrow, CSF: cerebrospinal fluid, KO: knock out, AD: Alzheimer's disease.
